# Effect of combining eight weeks of neuromuscular training with dual cognitive tasks on landing mechanics in futsal players with knee ligament dominance defect: a randomized controlled trial

**DOI:** 10.1186/s13102-022-00593-0

**Published:** 2022-11-22

**Authors:** Majid Hamoongard, Malihe Hadadnezhad, Ali Abbasi

**Affiliations:** 1 Department of Biomechanics and Sports Injuries, Faculty of Physical Education and Sports Sciences, Kharazmi University, Tehran, Iran; 2 Biomechanics and Corrective Exercise, Faculty of Physical Education and Sport Sciences, Kharazmi University, Hesari St, Tehran, Iran

**Keywords:** Dual-task training, 2D kinematics, Neuromuscular defects, Anterior cruciate ligament, Dynamic knee valgus, Knee

## Abstract

**Background:**

The performing of jump and landing in futsal simultaneous with divided attention is one of the most common mechanisms of non-contact anterior cruciate ligament (ACL) injury. Neuromuscular training has effectively reduced the risk of ACL injury, but the effect of neurocognitive training has received less attention. This study investigated the effect of combining 8 weeks of neuromuscular training with dual cognitive tasks on the landing mechanics of futsal players with knee ligament dominance defects.

**Methods:**

Thirty male futsal players (mean ± SD: age: 21.86 ± 3.27 years) with knee ligament dominance defects were purposefully identified by the tuck jump test and were randomly divided into the intervention and the control group. The intervention group performed dual task (DT) training for three weekly sessions for 8 weeks and 60 min each, while the control group only did activities of daily living. During the drop vertical jump test, 2D landing kinematics in two moments of initial contact (IC) and full flexion (FF) were assessed. Data were analyzed by means of 2 × 2 repeated measures ANOVA followed by post hoc comparison (Bonferroni) at the significance level of (α ≤ 0.05).

**Results:**

A significant improvement was observed in the intervention group compared to the control group for the dynamic knee valgus at IC (F_1,28_ = 6.33; *P* = 0.02, ES = 0.31) and FF (F_1,28_ = 13.47; *P* = 0.003, ES = 0.49), knee flexion at IC (F_1,28_ = 20.08; *P* = 0.001, ES = 0.41) and FF (F_1,28_ = 13.67; *P* = 0.001, ES = 0.32), ankle dorsiflexion at IC (F_1,28_ = 37.17; *P* = 0.001, ES = 0.72) and FF (F_1,28_ = 14.52; *P* = 0.002, ES = 0.50), and trunk flexion at FF (F_1,28_ = 20.48; *P* = 0.001, ES = 0.59) angles. Changes in the trunk flexion at IC (F_1,28_ = 0.54; *P* = 0.47, ES = 0.03) and trunk lateral flexion at IC (F_1,28_ = 0.006; *P* = 0.93, ES = 0.00) and FF (F_1,28_ = 2.44; *P* = 0.141, ES = 0.148) angles were not statistically significant.

**Conclusions:**

DT training compared to the control group improved landing mechanics in futsal players with knee ligament dominance defects.

*Trial registration*: Current Controlled Trials using the IRCT website with ID number IRCT20210602051477N1 prospectively registered on 20/06/2021.

## Background

In a national survey in the Netherlands, futsal, with an injury incidence of 55.2 per 10 000 h of sports participation, was among the ten sports with a higher injury rate [1]. Knee injuries are common among individuals participating in team sports, such as futsal [2]. Jumping and landing are the most common mechanisms of non-contact anterior cruciate ligament (ACL) injury [3]. More than one-third of non-contact ACL injuries in football frequently occur after landing from a jump [3]. The landing technique is complex and influenced by biomechanical, neuromuscular, and neurocognitive defects, so a stiff landing with minimum flexion in the trunk, hip, knee, and ankle causes a peak of the ground reaction force and increases the risk of ACL injury [4]. Inability to trunk control and hip movements can lead to increased internal rotation and adduction of the femur, eventually resulting in dynamic knee valgus (DKV) and increased forces acting on ACL [5]. Limitation of the ankle dorsiflexion range of motion during landing is also associated with decreased knee flexion in the sagittal plane and increased DKV in the frontal plane, which puts excessive tension on the ACL [6]. Sigward et al. [7] indicated that individuals with limited ankle dorsiflexion range of motion exhibited more DKV during landing.

In a sports competition, futsal players must perform several tasks simultaneously, including controlling the ball, decision-making, considering the opponent's movements, and performing sport-specific patterns [8]. These conditions cause more attention to cognitive stimuli and failure to follow the safe kinematic pattern during jumping and landing, which may expose a person to an ACL injury [9]. In fact, when the attentional demand for cognitive stimuli is greater than a certain amount due to the limited capacity of attention, the performance of other tasks performed simultaneously may be affected [10].

Studies examining the effects of more sport-specific cognitive tasks also appear to indicate that athletes exhibit more high-risk landing biomechanics when they must divide their attention (e.g., less hip and knee flexion, greater vertical ground reaction forces, and DKV) [11, 12]. Farvardin et al. [13] showed that adding a cognitive load to the kicking mechanics of futsal players in dual-task (DT) conditions reduces knee and hip flexion and more significant sagittal plane knee loading, which can increase the risk of ACL injury. Athletic performance is typically assessed under conditions associated with performing a sport-specific pattern simultaneously with decision-making and divided attention, known as DT [14]. In addition, it has been stated an athlete who is a deficit at DT may not be able to appropriately monitor neuromuscular control associated with the sport-specific tasks [9].

Neuromuscular defects (trunk, leg, quadriceps, and ligament dominance) are considered modifiable risk factors highly associated with ACL injury [15]. Knee ligament dominance deficit, or DKV, occurs when the motor control strategies adopted by the athlete do not provide sufficient dynamic stability for the knee joint. As a result, a significant portion of the ground reaction force during landing is absorbed by ligaments [16]. The tuck jump test consists of repeated plyometric activities that identify neuromuscular factors associated with ACL injury [17].

Neuromuscular training programs, including lower extremity strengthening, plyometric exercises, and proprioception training, are likely to reduce the risk of ACL injury by modifying movement patterns and pre-programmed feedforward strategies during jumps and landings [18]. Neuromuscular training aimed at reducing DKV angles and improving stability and coordination is considered important in ACL injury prevention [19]. Nagano et al. [20] reported that jumping and balance exercises positively affect knee mechanics. Despite various injury prevention programs, the incidence of ACL injury in athletes is increasing [21, 22]. Current training programs focus mainly on the individual's motor performance and do not consider cognitive challenges. Consequently, there is probably a gap between traditional injury prevention programs [23].

Neurocognitive factors such as reaction time, focus of attention, visual motor control, processing speed, and DT may influence injury risk via alterations to neuromuscular control during sport-specific tasks [21]. Evidence suggests that neurocognitive deficits play a key role in developing neuromuscular and biomechanical defects associated with ACL injury [24]. Likewise, athletes' poorer reaction time and processing speed have been identified as predictors of ACL injury [25]. Neurocognitive deficits can be considered modifiable risk factors that can be corrected with neuromuscular and cognitive training [4]. Neurocognitive approaches may be the missing link in ACL injury prevention exercises [26]. DT training is defined as performing two or more exercises (motor + cognitive) simultaneously [27]. Previous studies have shown that DT training leads to the development of new perceptual strategies, improvement of decision-making and attentional focus [28], enhanced retention and sustained attention [29], and finally, reduction of DT interference [27, 30]. To develop a valid training protocol for injury prevention, cognitive tasks should be considered, which may determine the limitation of the effectiveness of ACL injury prevention training programs [31]. The ability to maintain dynamic stability of the knee joint under conditions associated with cognitive challenges is crucial in preventing ACL injuries [10].

Adding cognitive tasks to neuromuscular training may improve lower extremity mechanics during jumping and landing [24]. A review by Moreira et al. [32] investigated the effect of DT training on the performance of athletes. It concluded that although DT training reduces performance in the short term, it improves working memory and attentional control in the long term. Sarulatha et al. [33] showed that progressive DT training improves motor function in older adults with balance disorders. DT and multi-task exercises can increase the capability of people to overcome the limited central nervous system processing capacity [34]. However, to our knowledge, no studies have targeted DT training to reduce the risk of ACL injury in athletes with neuromuscular defects.

This study aimed to investigate the effects of combining 8 weeks of neuromuscular training with dual cognitive tasks on the landing mechanics of futsal players with knee ligament dominance defects. Therefore, “does DT training improve landing mechanics in futsal players with knee ligament dominance defects?”. We hypothesized that a combined 8-week neuromuscular dual cognitive task training program would improve 2D kinematic measures of drop landings compared to a control group. The present study's findings can provide researchers with a new approach to ACL injury prevention training that targets the effect of cognitive tasks similar to competition conditions and can also increase the effectiveness of neuromuscular training.

## Methods

### Study design

Before data collection, this study was approved by the ethics committee of the Sports Sciences Research Institute with ID number (IR.SSRI.REC.1400.1071). Thirty male futsal players participated in this parallel-group randomized controlled trial (RCT) that was prospectively registered as a clinical trial with the code (IRCT20210602051477N1) in the Iranian Registry of Clinical Trials, date of first registration 20/06/2021. The allocation ratio (1:1) of the intended numbers of participants in each of the control and intervention groups. All participants were informed of the study procedures and signed an informed consent form prior to participating, per the Declaration of Helsinki.

### Participants

A priori power analysis (G*Power©, version 3.1.9.2, University of Dusseldorf, Dusseldorf, Germany) to obtain 80% statistical power with an alpha of 0.05, a beta of 0.20, and a medium effect size of 0.06, determined we would need 12 participants per group (a total sample size of 24 participants). Allowing a dropout rate of 10% and improved final statistical power, we enrolled 15 participants per group (total sample size of 30 participants). This effect size was comparable to previous research reporting changes in landing mechanics after neuromuscular training [18].

### Eligibility criteria

Inclusion criteria included futsal players with knee ligament dominance deficit identified by the tuck jump test [15], men in the age range of 18–30 years, and normal body mass index [35], history of participation in futsal in the past three years, 4 sessions per week [36], and not participating in injury prevention training programs in the last year. Subjects who showed DKV during the tuck jump test were identified as those with knee ligament dominance defects.

Exclusion criteria included a history of neuromuscular disorders, ACL injury or lower extremity and trunk injury requiring surgery during the last six months [37], visible malalignment in the lower extremity and trunk [10], or any medical disorder that affects the cognitive process and tasks [14], the subjects’ dissatisfaction and unwillingness to perform the research process, the lack of subjects contribution in two consecutive training sessions and three non-consecutive training sessions.

#### Randomization

Randomization was performed by an independent investigator unfamiliar with the testing protocol using a random allocation rule. The letters A and B were identified as markers for random groups assignment and were placed in sealed opaque envelopes in a box. Another researcher opened envelopes and proceeded with training according to the group assignment. These letters were numbered randomly selected and placed one after the other. Thus, the participants were divided into two groups A (intervention = 15) and B (control = 15). Group allocation was concealed using an opaque envelope until after athletes had been enrolled in the study to minimize potential bias. In the current study, a single-blind method was used where only participants were tried to be blinded from the study. For this purpose, the intervention and control groups went about their daily lives (futsal exercises, four weekly sessions). In addition, the intervention group also received DT training, and the control group continued futsal exercises without any information about the conditions of the other subjects.

The subjects were asked to perform an initial warm-up before the test to prevent injury. The pre-test warm-up protocol took 5 min and included squats with body weight (2 sets—8 repetitions), bipedal vertical jump (2 sets—5 repetitions), running, and dynamic stretching movements [10]. The post-test was conducted 1 week after the intervention for all subjects. The location of data collection and intervention group exercises for maximum application and generalizability was in the place of indoor soccer, especially for futsal players.

**Fig. 1:**
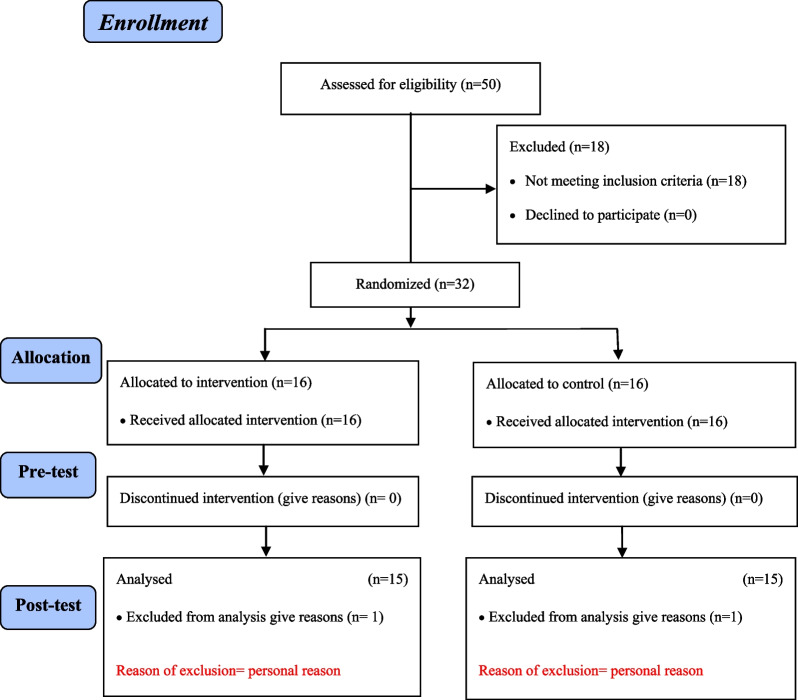
Consort flow diagram

Interventions included a combined 8-week neuromuscular training program with dual cognitive tasks performed simultaneously by the intervention group. After 8 weeks, a post-test was applied in pre-test conditions for both control and experimental groups, and the collected data were analyzed (Fig. 1).

### Outcome measurements

The current study used the tuck jump test to identify futsal players with knee ligament dominance defects (an increased reliance on frontal plane control compared to sagittal plane control). The drop vertical jump test, video camera, and kinovea software were used to investigate the landing mechanics.

2D kinematics with intraclass correlation-coefficient (ICC = 0.90–0.99) based on the Dingenen et al. [38] and the kinovea software (kinovea, version 0.8.15, USA), have been reported as valid and reliable [39]. In this study, two standard cameras (Samsung Galaxy Note 4, N910S, Korea) recorded drop vertical and tuck jump tests simultaneously. A camera was located on the sagittal plane, and a camera was placed on the frontal plane to record the subjects' movements at a distance of 3 m [15]. The cameras sat on a tripod perpendicular to the plane at a height of 65 cm and recorded the videos synchronously. Free video software (kinovea, version 0.8.15, USA) was used for recording videos [39]. Knee flexion, trunk flexion, and ankle dorsiflexion in the sagittal plane and trunk lateral flexion, and DKV in the frontal plane were measured.

2 × 2 (group × time) repeated measures ANOVA was used for each variable to compare the kinematic angles in the two moments of Initial Contact (IC) and Full Flexion (FF).

## Procedures

### Tuck jump test

The tuck jump test is a valid and reliable method with an intraclass correlation-coefficient (ICC = 0.94–0.96) to assess and diagnose neuromuscular deficits associated with an ACL injury [17]. Subjects were instructed to place their feet on the ground in the middle of a marked rectangle. This test involves performing continuous jumps with maximum height for ten seconds. Basic instructions given about how to perform the test included information about lifting the knees to hip height and trying to land on the same spot with their feet shoulder-width apart. Participants were not allowed to perform more than two tests before data collection. Each athlete carried out continuous tuck jumps on the designated spot after receiving basic instructions again about how to complete the trial. The knee ligament dominance was defined in the tuck jump test when knee valgus appeared at the landing and foot placement was not shoulder-width apart [15].

### Landing mechanics

**Fig. 2:**
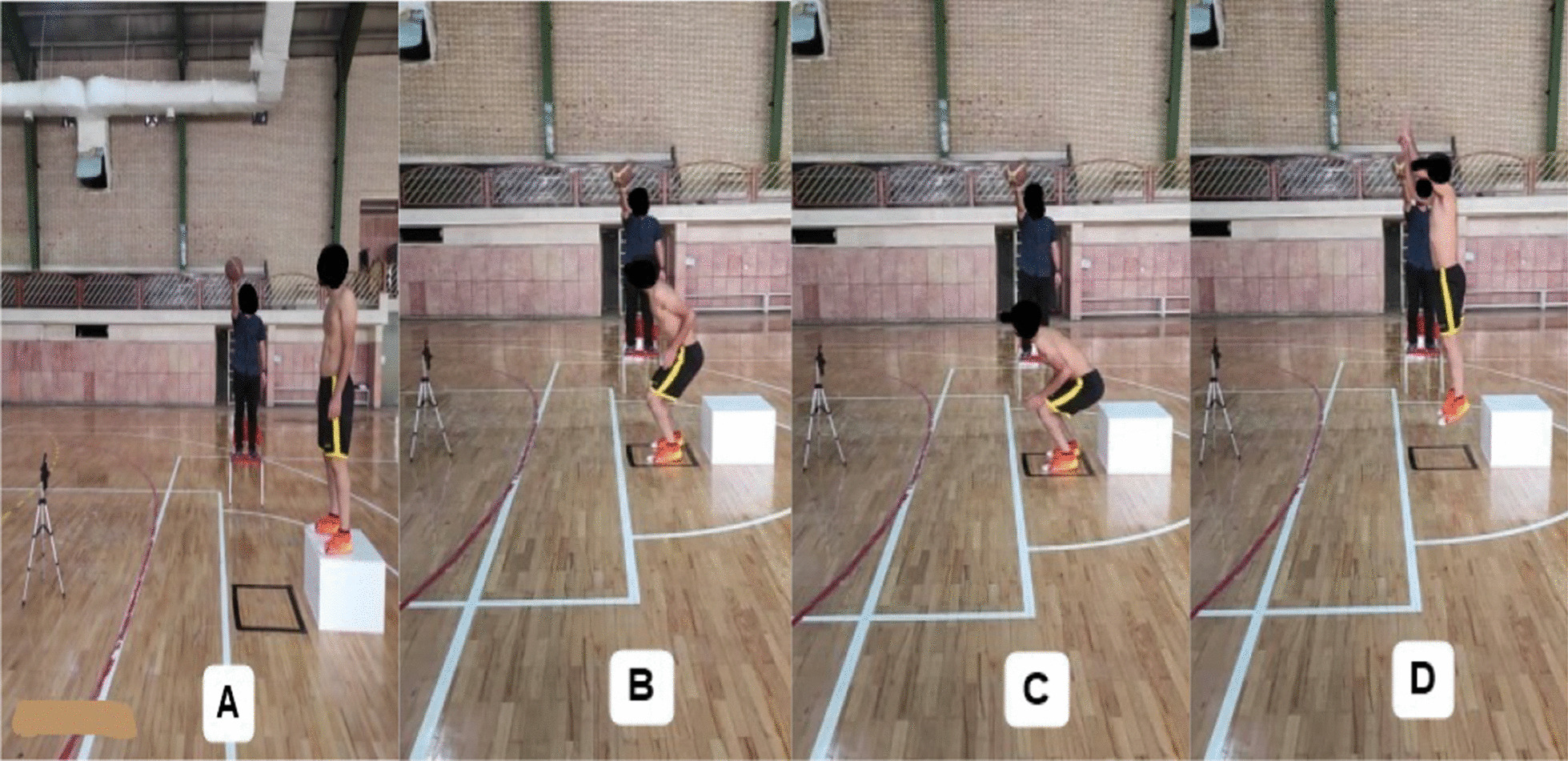
Drop vertical jump (DVJ): **A** Initial stance. **B** Initial contact. **C** Full flexion. **D** Max vertical jump

Biomechanical patterns at landing can be assessed during the drop vertical jump test [40]. The subject stood with his feet shoulder-width apart on a box of 40 cm in height. Subjects were instructed to drop directly down the box and immediately perform a maximum vertical jump; both raised their arms like a basketball rebound. The drop vertical jump has been proven valid and reliable, with a reliability coefficient greater than (0.93) [16]. Three successful jumps and a 30-s rest were performed between each jump. The trial was discarded and repeated if both feet did not land on the ground, if the athlete jumped off the box instead of dropping during the test. To standardize the jump height, a ball was placed above the subjects (Fig. 2). A baseline assessment of landing mechanics was completed for each participant upon enrollment.

### Neuromuscular training with dual cognitive tasks

Following completion of baseline testing, athletes in the intervention group participated in an 8-week progressive neuromuscular training program with dual cognitive tasks under the supervision of two experienced examiners. The neuromuscular training program based on those proposed by Ghanati et al. [18] consisted of 8 weeks of sport-specific training, agility, balance, neuromuscular coordination, strength training, proprioception, and plyometrics. Neuromuscular training programs include double-leg squats, walking lunges, single-leg squats, double-leg drop jumps, single-leg stance on an unstable surface, single-leg countermovement jumps, horizontal bounds, single-leg standing long jumps (Table 1) [18]. All squat exercises were performed with body weight, and a balance ball was used to stand a single leg on an unstable surface. This training protocol, program components, time, type, and repetition were recommended to prevent ACL injury. The neuromuscular training program consisted of three sessions per week during weeks 1–6 and two sessions per week during weeks 7–8 (22 sessions total). Training program interventions included 10 min of submaximal warm-up on a stationary bike, 40 min of exercise, 30 to 60 s of rest between each set, and 5 min to cool down. Finally, the total exercise time was between 45 and 60 min. The neuromuscular training program used in this study has been previously described and shown to reduce high-risk landing mechanics in healthy athletes [18]. It is important to note that all neuromuscular training sessions were performed simultaneously with cognitive tasks (DT training).

**Table 1:** Neuromuscular training program

Training	Week 1	Week 2	Week 3	Week 4	Week 5	Week 6	Week 7	Week 8
Double-leg squats	3 × 6	3 × 6	–	–	–	–	–	–
Walking lunges	3 × 6	3 × 6	–	–	–	–	–	–
Single-leg squats	3 × 6	3 × 6	4 × 8	4 × 8	4 × 12	–	–	–
Double-leg drop jumps	–	–	3 × 6	4 × 10	4 × 12	–	–	–
Single-leg stance, unstable surface	–	–	3 × 30 s	3 × 30 s	4 × 30 s	4 × 30 s	3 × 30 s	3 × 30 s
Single-leg countermovement jumps	–	–	3 × 6	3 × 8	4 × 8	4 × 10	3 × 8	3 × 6
Horizontal bounds	–	–	–	–	–	4 × 8	5 × 10	3 × 8
Single-leg standing long jumps	–	–	–	–	–	4 × 8	5 × 8	3 × 8

Sets × repetitions or seconds for each exercise across the 8-week neuromuscular training program

Athletes given 30–60 s of rest between sets

The cognitive tasks mainly included functional responses to sensory stimuli, verbal and visual memory, motor learning, speech, attention, and inhibition and were performed simultaneously with neuromuscular training [41]. Determining the ideal distance between stimuli to perform a DT was challenging. If the stimulus is faster than one to one and a half seconds, it was shown that cognitive error increases [42]. Therefore, using a two-second interval between stimuli is appropriate for healthy individuals in the present study; cognitive interventions with a time interval of 2 s were presented [42]. For instance, the subject must perform mathematics calculations simultaneously with all squat exercises.

It has been shown that more than three errors in neuromuscular control or one severe error indicate that the athlete is not ready to add neurocognitive challenges to neuromuscular training. Therefore, this criterion was used to control the training intensity [24]. Two experienced athletic trainers were used to implement the training protocol and the accuracy of performing cognitive tasks and monitoring the training process. The instructions were applied before each training session. In the first week, the external and internal focus of attention instructions were used to teach subjects the correct joint alignment and proper movement pattern. The full description of the neuromuscular DT cognitive training program is described in Table 2.

**Table 2:** Neuromuscular dual cognitive task training program

Neuromuscular training	Cognitive tasks simultaneous to neuromuscular training
Double-leg squats Walking lunges Single-leg squats	Forward and backward counting, naming months of the year forward and backward, Naming fish, dog or tree types, Participants were asked to remember pre-selected words and sing songs with the vocabulary included
Single-leg squats Double-leg drop jumps Single-leg stance, unstable surface Single-leg countermovement jumps	Reaction to the colors displayed verbally, simple mathematical problems, subtracting by twos from a number ≥ 30, visual and auditory stroop test, if the instructor says YES they respond NO and viceversa
Single-leg stance, unstable surface Single-leg countermovement jumps Horizontal bounds Single-leg standing long jumps	Participants will be asked to shift focus from a cognitive task to another on some of the dual tasks, if a green card is presented they have to say RED and when a red card is presented they have to say GREEN, Naming members of the family/clothes/professions

### Data analysis

To obtain 2D kinematic data, videos recorded by the 2 digital cameras at 30 frames per second were imported into kinovea software. The kinematic variables, including the articulated angles (DKV and knee flexion, ankle dorsiflexion, trunk flexion, and trunk lateral flexion) were extracted after modeling the lower extremities and trunk in this software. We placed reflective markers on the episternum, acromion process, anterior superior iliac spine (ASIS), great trochanter, medial and lateral epicondyles of the femur, medial and lateral malleoli [37], middle of the knee joint, and middle of the ankle joint [43] for 2D kinematic analysis.

The ankle dorsiflexion angle was the angle between the line formed by the lateral epicondyle of the femur and the lateral malleolus and a second line connecting the lateral malleolus and fifth metatarsal heads [43]. The trunk flexion angle was defined as the angle formed between the acromion and the greater trochanter and a line perpendicular to the ground. The trunk lateral flexion angle was defined as the angle between the line formed by both sides of the ASIS and the line formed by the midpoint of the ASIS and episternum. The knee flexion angle was defined by the angle between the line formed by the greater trochanter and lateral epicondyle of the femur and the line formed by the lateral epicondyle of the femur and lateral malleoli. DKV angle was computed using the angle between the line formed between the markers at the ASIS and the middle of the knee joint and the line formed from the markers on the middle of the knee joint to the middle of the ankle joint [37]. The middle of the ankle joint was defined at the midpoint of the medial and lateral malleoli markers, and the middle of the knee joint was defined at the midpoint of the medial and lateral femoral epicondyle markers [44]. Dingenen et al. [43] showed that measurements using the 2D kinematics method is valid and reliable and strongly correlate with data obtained by the 3D kinematics method.

2D kinematics were recorded at the two moments of IC and FF [3]. IC of the first landing phase was defined as the first frame in which ground contact was observed. In contrast, the FF angle was defined as the maximum angle between the femur and shank segments during the ground contact phase [45].

### Statistical analysis

To assess the normality of data distribution and homogeneity of variances, Shapiro–Wilk and Levene’s tests were used, respectively. Descriptive statistics were calculated for all variables, and mean, and standard deviation (SD) were reported. Independent samples t-test was applied to compare the demographic characteristics of the two groups. Then, according to the research design, Two-factor ANOVA test 2 (group: experimental, control) × 2 (time: pre-test, post-test) with a group x condition interaction was used to analyze the within and between group evaluation over the eight-week DT training. If a significant interaction effect was found between factors, post-hoc analyses (paired t -test) with Bonferroni adjustment for Pairwise comparisons were applied. Within group factor (pre-test to post-test) as a main effect of time and between-group as a main effect of the group were considered. Percentage changes from pre-test to post-test were calculated. Effect sizes (ES) using partial eta squared were calculated to increase the analysis power. ES were classified as small (0.01), moderate (0.06), and large (0.14) [46]. A modified intention to treat analysis based on the complete case method was used. In this method, since one person was randomly removed from each control and intervention group, they were excluded from the study. The analysis was performed only on those who completed the pre-test and post-test. Findings were analyzed at a significance level of 95%, with a statistical significance of (*P* < 0.05) and performed using IBM SPSS software (SPSS, version 26, Chicago; IL).

## Results

After completing the data collection form, the subjects (mean ± SD; age: 21.86 ± 3.27 years, weight: 68.91 ± 9.55 kg, height: 175.6 ± 6.49 cm, body mass index: 22.36 ± 2.29 kg/m^2^) were purposefully selected and randomly divided into intervention (n = 15) and control (n = 15) groups (Table 3). Two subjects were lost to follow-up due to personal reasons (control group, n = 1 and intervention group, n = 1). No adverse events were reported (Fig. 1). There was no significant difference between age (*P* = 0.27), weight (*P* = 0.63), height (*P* = 0.71), body mass index (*P* = 0.54), and sports history (*P* = 0.11) of both control and intervention groups (Table 3). Pre-test comparisons revealed no significant differences between groups at baseline testing for all 2D kinematic variables (*P* > 0.05) (Table 4). Therefore, the results of the Shapiro-Wilks and Levene’s tests confirmed that the data were normally distributed, and the variances were homogeneous (*P* > 0.05). All subjects participated in the pre-test and post-test after 8 weeks.

**Table 3:** Demographic and baseline characteristics of the participants (mean ± SD**)**

Variable	Total subjects (n = 30)	Control (n = 15)	Intervention (n = 15)	*P* value
Age (years)	21.86 ± 3.27	21.2 ± 2.80	22.53 ± 3.66	0.27
Weight (kg)	68.91 ± 9.55	69.57 ± 8.39	68.24 ± 10.85	0.63
Height (cm)	175.6 ± 6.49	174.73 ± 6.25	175.9 ± 6.88	0.71
BMI (kg/m^2^)	22.36 ± 2.29	22.64 ± 1.85	22.08 ± 2.70	0.54
Sport history (years)	5.83 ± 2.18	5.20 ± 2.14	6.46 ± 2.09	0.11

**Table 4:** Results of two-factor ANOVA test to compare the mean of kinematic variables

Kinematic Variable	Group	Pre-test Mean ± SD	Post-test Mean ± SD	F	*P* value	ES	Main effect of group		Main effect of time		Time* group Interaction effect	
							*P* value	ES	*P* value	ES	*P* value	ES
DKV at IC	Intervention	177.93 ± 5.92	182.86 ± 5.37	6.33	0.02*	0.31	0.45	0.02	0.10	0.09	0.01*	0.19
	Control	179.66 ± 5.67	178.60 ± 5.26	0.77	0.39	0.05						
DKV at FF	Intervention	174.46 ± 10.94	182.73 ± 9.74	13.47	0.003*	0.49	0.07	0.11	0.004*	0.25	0.18	0.06
	Control	170.33 ± 13.12	173.53 ± 9.66	1.20	0.29	0.07						
Knee flexion at IC	Intervention	136.60 ± 20.82	117.06 ± 10.33	20.08	0.001*	0.41	0.67	0.007	0.001*	0.34	0.01*	0.18
	Control	130.66 ± 10.31	126.60 ± 12.82	0.87	0.35	0.03						
Knee flexion at FF	Intervention	85.33 ± 12.74	74.73 ± 7.84	13.67	0.001*	0.32	0.001*	0.38	0.04*	0.13	0.004*	0.26
	Control	92.60 ± 9.71	94.86 ± 11.31	0.62	0.43	0.02						
Ankle dorsiflexion at IC	Intervention	92.06 ± 14.09	75.80 ± 6.78	37.17	0.001*	0.72	0.77	0.003	0.001*	0.36	0.002*	0.30
	Control	85.53 ± 12.42	84.33 ± 9.63	0.12	0.72	0.009						
Ankle dorsiflexion at FF	Intervention	67.00 ± 8.83	60.86 ± 7.23	14.52	0.002*	0.50	0.02*	0.16	0.002*	0.28	0.43	0.02
	Control	72.66 ± 9.82	68.87 ± 9.34	2.33	0.149	0.143						
Trunk flexion at IC	Intervention	25.80 ± 8.07	26.93 ± 7.22	0.54	0.47	0.03	0.18	0.06	0.37	0.02	0.08	0.10
	Control	24.53 ± 8.06	21.13 ± 8.39	2.96	0.10	0.17						
Trunk flexion at FF	Intervention	36.66 ± 7.67	43.80 ± 7.19	20.48	0.001*	0.59	0.14	0.07	0.36	0.03	0.001*	0.39
	Control	37.06 ± 13.84	32.46 ± 12.08	4.19	0.06	0.23						
Trunk lateral flexion at IC	Intervention	19.86 ± 4.12	19.80 ± 3.52	0.006	0.93	0.00	0.26	0.04	0.15	0.07	0.13	0.07
	Control	20 ± 4.45	22.53 ± 4.50	3.06	0.10	0.18						
Trunk lateral flexion at FF	Intervention	24.27 ± 6.02	22.07 ± 3.32	2.44	0.141	0.148	0.68	0.006	0.14	0.07	0.42	0.02
	Control	24.07 ± 4.89	23.40 ± 3.69	0.28	0.60	0.02						

*IC* initial contact, *FF* full flexion, *SD* standard deviation, *DKV* dynamic knee valgus, *ES* effect size

*Significant difference (*P* < 0.05)

As per Table 4, repeated measures ANOVA results revealed significant effects of the 8-week DT training. Significant group × time interaction effects were found for the DKV at IC (F_1,28_ = 6.77; *P* = 0.01; ES = 0.19), knee flexion at IC (F_1,28_ = 6.29; *P* = 0.01; ES = 0.18), knee flexion at FF (F_1,28_ = 10.07; *P* = 0.004; ES = 0.26), ankle dorsiflexion at IC (F_1,28_ = 12.17; *P* = 0.002; ES = 0.30), and trunk flexion at FF (F_1,28_ = 18.28; *P* = 0.001; ES = 0.39) angles. Additionally, significant main effects of time were found for the DKV at FF (F_1,28_ = 9.67; *P* = 0.004; ES = 0.25), knee flexion at IC (F_1,28_ = 14.65; *P* = 0.001; ES = 0.34), knee flexion at FF (F_1,28_ = 4.22; *P* = 0.04; ES = 0.13), ankle dorsiflexion at IC (F_1,28_ = 16.36; *P* = 0.001; ES = 0.36), and ankle dorsiflexion at FF (F_1,28_ = 11.24; *P* = 0.002; ES = 0.28) angles. The main effect of the group was significant at the knee flexion at FF (F_1,28_ = 17.42; *P* = 0.001; ES = 0.38), and ankle dorsiflexion at FF (F_1,28_ = 5.63; *P* = 0.02; ES = 0.16) angles.

Post hoc tests showed significant differences in the DKV at IC (F_1,28_ = 6.33; *P* = 0.02; ES = 0.31), DKV at FF (F_1,28_ = 13.47; *P* = 0.003; ES = 0.49), knee flexion at IC (F_1,28_ = 20.08; *P* = 0.001; ES = 0.41), knee flexion at FF (F_1,28_ = 13.67; *P* = 0.001; ES = 0.32), ankle dorsiflexion at IC (F_1,28_ = 37.17; *P* = 0.001; ES = 0.72), ankle dorsiflexion at FF (F_1,28_ = 14.52; *P* = 0.002; ES = 0.50), and trunk flexion at FF (F_1,28_ = 20.48; *P* = 0.001; ES = 0.59) angles in the intervention group compared to the control group. However, there was no significant difference between the pre-test and post-test in the control group. Changes in the trunk flexion at IC (F_1,28_ = 0.54; *P* = 0.47; ES = 0.03), trunk lateral flexion at IC (F_1,28_ = 0.006; *P* = 0.93, ES = 0.00), and trunk lateral flexion at FF (F_1,28_ = 2.44; *P* = 0.141, ES = 0.148) angles in the intervention group compared to the control group were not statistically significant (Table 4).

## Discussion

The present study aimed to investigate the effect of combining 8 weeks of neuromuscular training with dual cognitive tasks on the landing mechanics of futsal players with knee ligament dominance defects. The current study results demonstrated significant improvements in the DKV, knee flexion, and ankle dorsiflexion at both moments of IC and FF and trunk flexion at FF in the intervention group compared to the control group. However, changes in the trunk lateral flexion in both moments of IC and FF, and trunk flexion at IC in the intervention group compared to the control group was not statistically significant. The results supported the primary hypothesis statistically that a combined 8-week neuromuscular dual cognitive task training program would improve 2D kinematic measures of drop landings compared to a control group.

Previous studies have used DT training to improve the quality of life, gait, physical performance, and balance in the elderly [33, 47] and patients with musculoskeletal disorders [48]. Also, some studies have used DT to examine landing mechanics [10, 42]. Almonroeder et al. [11] concluded that adding cognitive tasks to jumping and landing activities reduced knee flexion angle and increased DKV and ground reaction force compared to alone jumping and landing training. This was the first study to examine the effect of DT training to prevent ACL injury in athletes with neuromuscular defects.

The human body is considered an inter-connected kinetic chain in which compensation movements or defects in one area cause functional defects in other parts of the body [49]. In fact, the movements of a joint are affected by the adjacent joints and affect them as well [50]. Biomechanical patterns that increase the DKV, decrease hip, knee, and trunk flexion, excessive trunk lateral flexion, and ankle dorsiflexion limitation increase the risk of ACL injury [5]. The limitation of the dorsiflexion range of motion prevents the ankle from achieving its closed-pack position and increases the probability of compensation movement patterns [51]. Decreasing trunk flexion in athletes increases the tension on quadriceps muscles to maintain the body's center of gravity and may cause ACL injury [5]. The trunk lateral flexion results in the displacement of the center of gravity and ground reaction force vector to the side, which in the form of kinematic chain reactions, compensates the knee in a dynamic valgus position [16]. Increasing the DKV angle leads to the athlete being near the position of no return, increasing the risk of ACL injury. In addition, Jumping and landing with the knee near full extension is a common mechanism of ACL injury [52].

Previous studies have shown that neuromuscular training improves the DKV and knee flexion angles and decreases the risk of ACL injury in people with biomechanical defects [53, 54]. Myer et al. [55] found that neuromuscular training positively influences lower extremities and thus prevents ACL injuries. However, there is a need to increase the effectiveness of these exercises. Despite comprehensive ACL injury prevention programs, ACL injuries occur considerably due to incorrect movement patterns [10], less compliance with injury prevention training programs to sports [8], and transfer deficiency from conscious awareness to automatic movements, unconscious and unpredictable sport-specific [56]. Athletes perform dynamic movements and cognitive processes simultaneously during the competition. Consequently, ACL injury prevention training programs should be similar to what happens in competition and cognitive challenges [14].

Fitts and Posner determined a model of motor learning that includes three stages: cognitive, associative, and automation, which can be used to learn how to add cognitive challenges to neuromuscular training [24]. The first step in learning is the cognitive stage skill, in which athletes learn how to perform movements and neuromuscular control properly [57]. Following the training progress, the individual enters a stage of associative in which cognitive challenges can be added to neuromuscular training [58]. The highest stage of learning a skill is the automation stage, in which a person can perform neuromuscular training with a safe movement pattern and without needing cognitive attention [57]. At this stage, difficult cognitive tasks can be combined with neuromuscular exercises [24]. Suppose athletes demonstrate good neuromuscular control in the cognitive stage. In that case, neurocognitive challenges (e.g., DT) can be added to their training to assess their readiness to progress to associative, and automation stages [24]. DT is related to two theories of automatic and controlled information processing. Automatic processing is fast and spontaneous, whereas controlled processing is slow and requires working memory and attention control [32]. Probably, DT training improves the transfer of motor skills from the control stage of information processing to the automatic stage [24, 32]. It has been shown that DT training in children improves the transfer effect in the cognitive domain [59].

DT training involves a combination of neuromuscular training with cognitive tasks that increase the subjects' ability to overcome processing limitations in the central nervous system, ultimately leading to the transfer and automation of movement patterns [32, 59]. Training with DT increases the excitability of the brain's dorsolateral prefrontal cortex, which is associated with performing DT, and may reduce the interference of these tasks in competition conditions [30]. Wollesen et al. [59] showed that DT training might improve cognitive and motor performance in children and adolescents. Likewise, Jaiswal et al. [60] revealed that 4 weeks of DT training improves static and dynamic balance, proprioception, pain, strength, and ankle range of motion in participants with chronic ankle instability. In contrast, Cakir et al. [61] investigated the effects of 6 weeks of DT training on physical fitness. They concluded that DT training might improve postural control and does not significantly affect lower limb power.

Given the findings, the neuromuscular dual cognitive tasks training program improved some kinematic indicators (DKV, knee flexion, ankle dorsiflexion at IC and FF, and trunk flexion at FF) landing, which can significantly decrease the risk of ACL injury in athletes. Indeed, DT training had a more effect on the lower limbs' kinematic variables. Reduced ankle dorsiflexion, trunk and knee flexion in the sagittal plane, and increased DKV and trunk lateral flexion in the frontal plane may collectively increase the risk of ACL injury [5]. Aerts et al. [54] showed that neuromuscular training significantly improves maximal knee and hip flexion, and trunk flexion and reduces the DKV angles during jumping and landing. However, this study used only jump and landing exercises.

Regarding not observing significant differences in trunk flexion at IC and trunk lateral flexion at IC and FF angles, it has been shown that core stability training improved trunk neuromuscular control in athletes [62]. One of the possible reasons for the difference in the results can be stated that most of the mentioned studies targeted participants with trunk dominance defects and used special training for core stability [62, 63]. Therefore, changes in the subjects were more significant, while the subjects of the present study were individuals with knee ligament dominance defects. Hence, it can be concluded that core stability exercises are more effective in trunk neuromuscular control compared to DT training. In addition, it is possible that the test was not challenging enough to show differences. Consequently, the researcher guessed that the differences would probably be more obvious if the single-legged drop jump test had been used instead of double-legged drop vertical jump test. The low sample size was another possible reason.

We believe that the results of our study provide valuable insight into the effects of DT training in athletes with neuromuscular defects. It is recommended that coaches, physiotherapists, and rehabilitation specialists should use DT training to improve safe landing mechanics and decrease the risk of ACL injury in futsal players with biomechanical and neuromuscular defects. As in all studies, limitations arise that can affect the results. To our knowledge, this was the first study to investigate landing mechanics during drop vertical jump tests before and after neuromuscular training simultaneously with dual cognitive tasks. Therefore, comparing our results with existing literature was difficult. First, our study only included male futsal players at high risk of ACL injury identified by the tuck jump test. As a result, we cannot generalize our findings to female athletes and patients with ACL injuries. Second, is the lack of using 3D kinematics as a gold standard for assessing landing mechanics. Third, we did not use another group to do neuromuscular training alone. Therefore, we cannot determine if the changes observed in our subjects were due to the neuromuscular training or the emphasis on adding cognitive tasks. Another limitation is that the dual cognitive task included in the DT training (e.g., Forward and backward counting, naming months of the year, simple mathematical problems) is not relevant to futsal. Probably, the use of special DT training will lead to better transfer and retention. For instance, cognitive training can involve the athlete paying attention to teammates' movements instead of simple mathematical problems. Finally, if drop vertical jump, including additional cognitive demands, was used to assess the landing mechanics, the sport-specific tasks were better simulated.

## Conclusion

Neuromuscular training with dual cognitive tasks (DT training) improved landing mechanics (dynamic knee valgus, knee flexion, and ankle dorsiflexion in both moments of IC and FF, and trunk flexion at FF angles) in futsal players with knee ligament dominance defects. It is recommended that rehab trainers should use DT training to decrease the risk of ACL injury in futsal players.

## Data Availability

The datasets used and/or analyzed during the current study are publicly available from the corresponding author upon reasonable request.

## Abbreviations

ACL: Anterior cruciate ligament
ES: Effect size
IC: Initial contact
FF: Full flexion
ASIS: Anterior superior iliac spine
SD: Standard deviation
DKV: Dynamic knee valgus
RCT: Randomized controlled trial
DT: Dual-task
